# Spinal Fusion for Chronic Low Back Pain: A ‘Magic Bullet’ or Wishful Thinking?

**Published:** 2016-03

**Authors:** KS Dhillon

**Affiliations:** KPJ Selangor Specialist Hospital, Shah Alam, Selangor, Malaysia

**Keywords:** Chronic low back pain, spinal fusion

## Abstract

Chronic low back pain is a common, disabling and costly health problem. The treatment of chronic low back is difficult and is often ineffective. For treatment to be effective the cause of the pain has to be established but unfortunately in 80% to 95% of the patients the cause cannot be determined despite the existence of modern imaging techniques. A pathoanatomical diagnosis which fits into a classical disease model where successful treatment can be carried out, can only be made in 5% to 7% of the patients. The back pain in the rest of the patients where no pathoanatomical diagnosis can be made is often labelled, unscientifically, as chronic low back pain. Despite the existence of sophisticated imaging techniques and a plethora of diagnostic test the source of pain in patients with nonspecific back pain cannot be established. There exist no causal relationship between imaging findings of degenerated disc, lumbar facet arthritis, spondylosis, spondylolysis and spondylolisthesis, to the pain in these patients. Surgical treatment of non-specific back pain where no pathoanatomical diagnosis has been established is bound to fail. Therefore the outcome of spinal fusion in these patients can be no better than nonsurgical treatment. Spinal fusion is a major surgery which can be associated with significant morbidity and occasionally with mortality. Yet there is rapid rise in the rates of spinal fusion. There is a growing tension between ethics and conflicts of interest for surgeons. The spine, unfortunately, has been labelled as a profit centre and there are allegations of conflicts of interest in the relationship of doctors with the multi-billion dollar spinal devices industry. The devices industry has a significant influence on not only research publications in peer review journals but also on decisions made by doctors which can have a detrimental effect on the welfare of the patient.

## Introduction

Low back pain represents a common disabling and costly health problem. Unfortunately the cause of back pain can be accurately diagnosed and treated in only a small proportion of the patients, where specific spinal pathology such as tumours, infection, fractures and nerve root pain caused by prolapsed disc or spinal stenosis, is present. In vast majority of the patients the cause of the pain is not known and such pain has been unscientifically labelled as non-specific back pain. A pathoanatomical diagnosis cannot be made in these patients, even with modern diagnostic imaging techniques such as magnetic resonance imaging of the spine, and this makes the treatment of non-specific back pain difficult. Surgeons are treating the symptom back pain and not a disease, with surgery, when they offer spinal fusion as a modality of treatment to such patients. This is partly due to an ‘orthopaedic surgery lacuna’ which is caused by the nature of training received by orthopaedic or spinal surgeons. Surgeons are trained to treat conditions which are amenable to surgery and are not trained to treat patients who do not need surgery unlike the neurologists and cardiologists who are trained to treat patients who do not need surgery. This ‘surgical lacuna’ has led many surgeons to believe that spinal fusion surgery will cure the patient of chronic low back pain and this is partly responsible for an exponential increase in rates of spinal fusion around the world. Besides this, there is a strong element of conflicts of interest which has caused a spike in spinal fusion rates. When surgery is done for a symptom and not for a disease the outcome tends to be poor and this increases the risk of negligence suits. It is common knowledge that litigations in this arena in Malaysia is on the rise. Is there justification for spinal fusion in the treatment of patients with non-specific low back pain?

### Definition and classification of chronic low back pain

A review of the literature reveals that there is no uniform definition for chronic or recurrent low back pain although a uniform definition is essential for the study of prevalence and treatment outcome of low back pain. There appears to be some consensus that low back pain which persists for at least 12 weeks can be classified as chronic low back pain and that which is present for less than 6 weeks can be classified as acute back pain. Back pain that last between 6 weeks to 12 weeks is often classified as subacute back pain. Repeated episodes of back pain cannot be classified as chronic back pain although such bouts of back pain have been present for many years unless each episode of back pain last for at least 12 weeks. An episode of back pain is defined as back pain lasting for at least 12 hours and recurrent back pain would be defined as the presence of least two such episodes in a year^1^.

Waddell’s classification of low back pain is widely accepted though there are several available classification for low back pain. Waddell’s diagnostic triage divides low back pain into three categories;

- Specific spinal pathology which can be found in 1% to 2% of patients. This would include diagnoses such as tumours, infections, fractures and cauda equina syndrome.
- Radiculopathy caused by disc prolapse and spinal stenosis which is seen in about 5% of the patients.
- Non-specific low back pain which occurs in about 85 to 95% of the patients^2^.

In this diagnostic triad, the first two categories encompass a proper pathoanatomical diagnosis which fits into a classical disease model and this makes effective treatment possible. On the other hand with the third category, the absence of a pathoanatomical diagnosis makes effective treatment fraught with difficulties. Waddell has eloquently described a diagnosis of non-specific backache as ‘intellectually and scientifically inadequate and it fails to provide any biological basis for real understanding’ which results in treatment remaining ‘empirical or based on unproven hypotheses’^2^. The belief that diagnoses such lumbar strain or degenerative spine disease causes chronic low back pain remain unfounded and this leaves a lot of room for uncertainty about treatment, prognosis and clinical outcome^2^.

### Pathoanatomical diagnosis of non-specific low back pain

Spinal fusion, for progressive or unstable spondylolisthesis, spinal trauma, tumours and spinal infections, has a well-established role and the outcome is good because there is a pathoanatomical diagnosis. On the other hand in patients with non-specific back pain where no pathoanatomical diagnosis exit and the cause of back pain is not known the value of spinal fusion remains questionable. Eliciting the cause of back pain in patients with chronic non-specific back pain remains a dilemma.

The degenerated intervertebral disc is most often implicated as the cause of pain in patients with non-specific low back pain. Such pain is usually referred to as discogenic back pain. Disc degeneration leading to abnormal shock loading of the disc and micro trauma to annulus and the endplate is believed to cause the pain. This trauma to the annulus and the endplate also allows blood vessel and nerve ingrowth into the normally avascular and aneural disc^3^. The disc is implicated in about 40% of the patients with non-specific low back pain^4^. The facet joints is believed to be the source of low back pain in 15 to 40% of the patients^5^ while the sacroiliac joint is implicated in about 15% of the patients^6^. Though we believe that these three are the main sources but not the only source of chronic low back pain, no conventional clinical test can discriminate the source of pain in patients with disc, facet joint or sacroiliac joint abnormalities^4-6^.

A simple relationship of radiographic structural abnormalities of the lumbar spine and low back pain cannot exist because many individuals with such structural spinal abnormalities are asymptomatic^7^. Systematic review of published studies show that there is a lack of firm evidence for a causal relationship, between radiographic findings of degeneration of the spine as defined by disc space narrowing, osteophytes and sclerosis, and non-specific back pain. Neither does a causal relationship exist between radiographic evidence of spondylosis, spondylolysis, spondylolisthesis, spina bifida, transitional vertebra nor with Scheuermann’s disease and non-specific back pain^8^.

Modern imaging techniques can now allow us to accurately depict the anatomical changes that occur with the degeneration of the disc. However, the clinical significance of these changes depicted on magnetic resonance imaging (MRI) remains elusive and often confusing^9^. In asymptomatic adults, degeneration of the disc can be seen in about 40 to 80% of individuals and it increases with age, disc protrusion can be seen 40 to 70%, end plate changes in 10 to 30% and annular disruption in 25 to 70% of adults who are asymptomatic^10^. Jansen *et al*^11^ in a study of 98 asymptomatic individuals found that an MRI examination of the spine revealed a normal disc in only 36% of the individuals. Fifty two percent had a disc bulge at one level, 27% had disc protrusion and 1% had disc extrusion. 19% had Schmorl’s nodes, 14 % had annular tears and facet arthropathy was present in 8% of the subjects. The findings were the same in males and females. The high prevalence of these findings in asymptomatic individuals and a high prevalence of back pain in general population suggest that the MRI findings of bulges or protrusions in people with low back pain may frequently be coincidental. Hence it makes sense that abnormalities on magnetic resonance images must be strictly correlated with age and any clinical signs and symptoms present before surgery is contemplated.

Since anatomical diagnostic tests such as radiographs and magnetic resonance imaging the gold standard are of not much help in elucidating the cause of non-specific low back pain, can other tests help in making a decision as to which of the patients with non-specific low back pain will benefit from surgery? Discography has in the past been advocated by some proponents as a useful decision making tool.

### Lumbar discography

Discography is used to determine if the low back pain the patient is experiencing is caused by disc pathology. In this procedure dye is injected at pressure of between 15-25 psi, under fluoroscopy, into the suspected disc while the patient is sedated. If the injection reproduces the pain at the same site that the patient has been experiencing before the procedure then it is believed that the disc is the source of the patient’s low back pain (positive discogram). However there is no universally accepted definition as to what a concordant pain response is and there is a lack of reliability studies on discography^12^. It is important to know whether discography can accurately define the disc that is generating the patient’s pain for it to be any use in spinal surgery.

We know that imaging of the spine with scans can reveal the degenerated morphology of the disc but the scans cannot tell us that the disc is the source of the pain. This is obvious from the fact that the imaging morphology of the disc does not change over short periods of time but the patient’s symptoms do. Can a discogram which is an invasive procedure with potentially serious complications such as discitis, provide us with the information needed regarding the source of the patient’s pain?

Sackett and Hayes^13^ (2002) have proposed that a critical test of validity of a diagnostic procedure involves measuring it against a gold standard in a clinical setting. The test should be able to distinguish between patients with and those without the target disorder or low back pain and furthermore patients who undergo the test should fare better compared to those patients who did not have the test. Unfortunately there is no reasonable gold standard against which to test discography. Despite the lack of proven validity against a gold standard, discography has been used to recommend spinal fusion in patients with non-specific low back pain.

Carragee*et al* ^14^ (2006) did a study to test the hypothesis that positive provocative discography will accurately identify patients with low back pain due to a primary discogenic lesion and a clinical cure will be achieved in such patients with a successful spinal fusion. This prospective study was carried out between 1996 and 2000. The first cohort of 32 patients had an episode of low back pain for 6 to 12 months which did not respond to conservative treatment and the discogram was positive at one level with a normal discogram at adjoining levels. The second cohort included 34 patients with an unstable spondylolisthesis who did not respond to conservative treatment. Patients with selection comorbidities such as compensation claim, abnormal psychometric test, occupational disabilities and prior lumbar surgery were excluded. The patients were followed up for 2 years.

When a high level criterion of successful outcome (highly effective) was adopted, 71.9% of patients in the spondylolisthesis group had a successful outcome after spinal fusion and in the discogenic group only 26.6% had a successful outcome. When a low level criterion of successful outcome (minimal acceptable outcome) was used 91.7% of the spondylolisthesis group had a successful outcome as compared to 43% in the discogenic group. After ‘adjusting for surgical morbidity and drop out failure, by either criteria of success, the best case positive predictive value of discography was calculated to be 50% to 60%’^14^. The study showed that provocative discography is not highly predictive in identifying intradiscal lesions as a cause of chronic low back pain. The usefulness of the test hence remains to be proven.

False positive findings on discography are also common. Carragee *et al*^15^ (1999) did an experimental disc injection in patients who had no past history of low back pain but developed back or buttock pain after posterior iliac graft harvesting for non-thoracolumbar surgical procedures. They studied 8 subjects who had 24 disc injections and 14 disc injections produced some pain response, 35.7% produced non-concordant pain, 50% produced similar pain and 14.3% of the injections produced the ‘exact’ pain. By the usual criteria for positive discography, 50% would have been classified as positive in this group of patients who had no back pain prior to the graft harvest. The response of concordant pain on discography appears to be less meaningful than is often believed.

Systematic review of accuracy of other tests such as orthosis immobilization and temporary external fixation of the spine to identify patients with chronic low back pain for whom spinal fusion is a predictable and effective treatment has not proved to be useful^16^. Can spinal fusion be an effective procedure for treatment of chronic non-specific low back pain when there are no accurate diagnostic tests to identify patients who will benefit from such treatment?

### Outcome of spinal fusion for non-specific low back pain

None of the imaging studies or other diagnostic test is able to accurately localise the source of pain in patients with non-specific low back pain. How can we then expect lumbar fusion to effectively treat the patient’s pain? Yet there has been a rapid increase in fusion rates (336%) of the lumbar spine from 1996 to 2001in the United States of America^17^. In England there has been an almost direct relationship between the numbers of operations performed per year and number of orthopaedic and neurosurgeons per head of population^18^. Do the results of spinal fusion justify the increase in fusion rates or are there some conflicts of interest?

Fairbank *et al*^19^, in 2005, published the results of a randomised controlled trial which assessed the clinical effectiveness of spinal stabilization or fusion compared to intensive rehabilitation for patients with chronic low back pain. Their cohort included 349 patients between the ages of 18 to 55 years with at least one year of low back pain who were considered by an experienced surgeon that they were candidates for spinal fusion. The patients were randomised into two groups, 176 to the surgical group and 173 to the rehabilitation group. The cohort was followed up for 2 years. At 2 years the clinical outcome was assessed using the Oswestry low back pain disability index, which is scored 0% (no disability) to 100% (totally disabled or bed ridden) and is designed to assess the limitation of activities of daily living. Other assessments included the shuttle walking test, short form (SF) general health questionnaire, and psychological distress and risk assessment (DRAM).

The mean Oswestry disability index changed favourably from 46.5 to 34 in the surgical group and from 44.8 to 36.1 in the rehabilitation group. The mean difference between the groups was about -4.1 in favour of the surgical group. There was no difference between the groups in the shuttle walking test and other outcome measures. There were surgical complications in 19 patients. Eleven patients in the surgical group had reoperations. Complications included dural tears, excessive bleeding, implant problems, fractures and vascular injury.

The study showed that there was no clear evidence that primary spinal fusion surgery was any more beneficial than intensive rehabilitation in patients with chronic low back pain. Surgery while not having any superiority over conservative management was associated with potential risk and increased cost.

Brox *et al*^20^ (2010) did a 4 years follow up of patients to compare surgical versus non-surgical therapy in the treatment of chronic low back pain. In this study of two merged randomised clinical trials the authors compared instrumented transpedicular fusion with cognitive intervention and exercises in 124 patients who had disc degeneration and one year of symptoms. This study included some patients who had previous surgery for disc herniation while others had no previous spinal surgery. Of the 124 patients 66 patients were assigned to the surgical group and 58 to the non-surgical group.

The study showed that lumbar fusion was not superior to cognitive intervention and exercises at reliving back pain, improving function and return to work at 4 years. However there were 14 patients (24%) randomised to the non-surgical group who underwent subsequent surgery (non- adherence to protocol) while 15 patients (23%) in the surgical group had to undergo reoperation.

These studies lack placebo controls. The improvements in the surgical group may be placebo mediated. Hence it is not possible to know whether the marginal positive outcome in these patients reflect the natural course of the disease, placebo effect, patient expectation, or the care provided^20^. It is a fallacy to believe that new technical solutions in the hands of a skilled surgeon will provide faster and greater improvements in the patient’s symptoms unless the solutions are based on sound knowledge backed by good randomised studies. At the present time there is insufficient evidence to determine the effect of fusion compared to non-surgical treatment. Methodological limitations of the published randomised trials prevent any firm conclusions to be drawn on the effectiveness of spinal fusion for chronic low back pain^21^.

The Agency for Healthcare Research and Quality (ACHR), U.S. Department of Health and Human Services which conducts the effective health care program as part of its mission to organise knowledge, has conducted an extensive comparative effectiveness review of spinal fusion for treating painful lumbar degenerated discs or joints and has come to the conclusion that there is insufficient evidence to determine the benefits of lumbar fusion compared to more intensive rehabilitation programs^22^.

In the absences of sufficient evidence of benefits of spinal fusion for chronic non-specific low back pain, is it safe to recommend the procedure to patients?

### Complications of spinal fusion

Spinal fusion is a major surgical procedure which involves extensive dissection, decortication of bone, blood loss and longer operating time. Often implants have to be used. More extensive and prolonged procedures are usually associated with more complications. There is a tendency among spinal surgeons to correct all anatomical abnormalities to prevent future symptoms, leading to more complex fusions, although there is no evidence that such ‘prophylactic’ surgery has any benefits. Complex fusions include multi-level surgery involving multiply approaches.

Deyo *et al*^23^ (2010) did a study to evaluate major complications in Medicare patients undergoing surgery for spinal stenosis in the United States in 2007. Their study of Medicare data avoided the bias which is often associated with studies by surgeons of selected patients from select centres. They were able to obtain nearly complete data on repeat hospitalization and mortality.

They studied 32,152 patients who had surgery for spinal stenosis in the first 11 months of 2007. The patients were 66 years and above. They analysed major medical complications, wound complications and the 30 day mortality. The major medical complications included those that needed cardiopulmonary resuscitation or repeat endotracheal intubation and mechanical ventilation due to cardiorespiratory arrest, acute myocardial infarct, respiratory failure, pulmonary embolism, pneumonia and stroke. The wound complications included haemorrhage, haematoma, seroma, wound break down and post-operative infections. Mortality included all deaths within 30 days of hospital discharge.

In patients who had complex spinal fusion, there was a 5.6% rate of major complication and the 30 day mortality was 0.6%. Complex fusions had an odds ratio of 2.95(95%CI) for life-threatening complications compared to decompression without fusion. The re-hospitalization rate was 13% in patients who had complex fusions. The wound complications were also higher in patients who had complex procedures.

In a study by Fritzell *et al*^24^ (2001) comparing surgical with non-surgical treatment for low back pain 18% of the patients in the fusion group developed early (within 2 weeks) complications and 6% had late complications. Complications included, bleeding, neural injury, heart failure, major GI bleeding, pulmonary oedema, aspiration sepsis, pulmonary embolism, dural tears, haematomas, pseudoarthrosis and even wrong level surgery. In patients who had complex fusion the complication rate was 31%. The reoperation rate in the surgical group was 6%.

Brox *et al*^25^ (2003) in a study comparing instrumented lumbar fusion with conservative treatment for chronic low back reported a 18% complication rate in the surgical group. In the Fairbank^19^ (2005) study the complication rate in the surgical group was 10.7% and the reoperation rate was 0.6%. In the 2010 Brox *et al*study^20^ the reoperation rate in the surgical group was higher at 23%.

There are wide variations in the complication and the reoperation rates in these studies because of insufficient reporting and variations in surgical techniques. This makes it difficult to determine conclusively the complication rates of lumbar fusion in these patients^22^. Nevertheless, complications associated with spinal fusion are not uncommon and can be life-threatening.

Despite a lack of superiority of spinal fusion over non-operative treatment of patients with chronic non-specific low back pain, there has been a steep rise in the rates of spinal fusion over the last two decades. Spinal fusion is also associated with morbidity and mortality. This begs the question as to why there is such a trend.

### ‘Spine as a profit centre’

There is growing tension between ethics and conflicts of interest with some surgeons becoming less altruistic and allegations of unethical behaviour among doctors becoming more rampant. The American press is often inundated with reports of unethical behaviour among doctors and these include accusations of exorbitant professional charges, royalties paid to doctors without intellectual property and marketing of medical products and services by doctors for economic gain. Spinal fusion for chronic low back is one such service. Spinal fusion is one of the most lucrative areas of medicine and it generates billions of dollars for the hospitals and the surgeons^26^.

In the year 2001 in the United States, 122,000 lumbar fusions were carried out for degenerative disease of the spine and this represented a 220% increase from 1990. This increase became more obvious after 1996 when fusion cages for spinal fusion became available. The increase in lumbar fusion from 1996 to 2001 was 113%, while for hip and knee arthroplasty it was only 13% and 15% respectively^27^. Studies show that a higher proportion of fusion procedures and the introduction of new spinal implants between the years 1993 to 1997 did not reduce re operation rates. In fact the reoperation rates were higher in the late 1990’s as compared to the early 1990’s^27^. The authors were of the opinion that introduction and marketing of new surgical devices and the influence of key opinion leaders is the likely reason for invasive procedures in the absences of new indications. Other possible reasons being financial incentives to hospitals and surgeons as well as the desire of surgeons to be innovators.

The influence of key opinion leaders and financial incentives for surgeons has hit the headlines in major U.S. newspapers in recent years. Allegations of kickbacks to spine surgeons to use their products, relationship of surgeons to biomedical firms with financial arrangements involving multibillion dollar medical devices industry have been highlighted^28^.

One such debacle was that of the use of recombinant human bone morphogenetic protein-2 (rhBMP-2), a bone growth factor for spinal fusion. The Spine Journal, June issue 2011, gained attention from surgeons, researchers, patients, media, and industry when it focused attention on the controversial rhBMP-2 synthetic bone growth factor for use in spinal fusion surgery. It highlighted the limitation of industry sponsored research, bias in research development and reporting as well as weaknesses of peer review publications and inadequate disclosures and ethical shortcomings. The industry sponsored doctors involved in the promotion of rhBMP-2 through publication of studies in peer review journals which showed no complications with the use of rhBMP-2, received millions of dollars in royalties from Medtronic, the manufacturer of the product^29^. Subsequent non-industry studies showed that the use of rhBMP-2 was associated with many complications^30^.

Conflicts of interest through consulting ties and other relationship with device manufactures aside, doctors have now started becoming investors in spinal implant manufacture and distribution. As of Oct 2012, there were at least 20 states in U.S. with multiple active physician owned distributorships (PODs) which supply devices to hospital, with California alone having 40 such distributorships^31^. This has sparked fears that this would provide extra financial incentive for surgeons to recommend spinal fusion.

This prompted the Congress in the U.S. to ask the U.S. Department of Health and Human Services to investigate the prevalence and use of spinal devices supplied by physician-owned distributorships (PODs)^32^. In 2011, PODs supplied devices used in one in five spinal surgeries billed to Medicare and contrary to claims by such distributorships the cost were not lower. A third of hospitals surveyed purchased devices from PODs. After hospitals started buying devices from PODs, the rates of spinal fusions grew faster in hospitals that bought devices from PODs compared to fusion rates in hospitals overall. In 2012, surgeons did more spinal surgeries at hospitals that purchased spinal devices from PODs than at hospitals that did not get their devices from PODs.

This new and growing area of partnership between surgeons and the device manufacturers has led some surgeons to express concern about such partnerships, because, they believe that it is unethical and it will bias the doctor’s choice of what is best for the patient. Others have described such partnerships as ‘an awfully pernicious conflict of interest’ for doctors^26^. Some have gone further to described this new business model as ‘low hanging fruits’ waiting to be plucked and not to be deprived of the opportunity Malaysian surgeons have jumped on the bandwagon^33^.

## Conclusions

There appears to be some consensus now regarding the definition of chronic low back pain though there are various definitions in published studies. Uniformity of definition will provide a basis for effective study of the prevalence and outcome of treatment of patients with chronic non-specific back pain.

Unfortunately in patients with chronic low back pain a specific pathoanatomical diagnosis can only be made in about 5% to 7% of the patients where treatment can be effective. In about 85% to 95% of the patients with chronic low back pain a pathoanatomical diagnosis cannot be made and this makes effective treatment difficult. In such patients non-specific chronic back pain is a symptom and not a disease. Logically a symptom cannot be treated by surgery.

Degenerated disc is usually implicated as the cause of chronic low back pain in most of the patients while in others the lumbar facet joints and the sacroiliac joints are believed to be the cause of chronic low back pain. Anatomical changes of degenerative pathology of the spine which are accurately depicted by MRI scans of the spine have no causal relationship to the patient’s symptoms. Even provocative discography which has been touted as a valid diagnostic test in the past has failed to live up to its expectation. Despite these drawbacks in our ability to make an accurate clinical diagnosis, there has been a steep increase in the number of patients who are treated with spinal fusion for non-specific low back pain over the last two decades. Chronic non-specific low back pain is a symptom and not a disease, hence logically surgery cannot be an effective mode of treatment for non-specific low back pain.

Good quality medical literature which compares the clinical outcome of patients with chronic low back pain who were treated conservatively, with those treated with spinal fusion does not exit. There are two level 1 studies with a two to four years follow up which did such a comparison but these studies did not show superiority of surgical treatment over nonsurgical treatment. There is no doubt that spinal fusion can be associated with significant and sometimes serious medical complications including mortality.

Despite the absence of good evidence to support the efficacy of spinal fusion for chronic low back pain, the rates of spinal fusion has rocketed in recent decades. This has raised concerns about conflicts of interest and unethical behaviour among healthcare providers. Accusations of kickbacks to spine surgeons from the medical devices industry have made headlines in major newspapers in the US in recent years. Another area of concern is the involvement of spine surgeons in the manufacture and distribution of spinal implants and this has created pernicious conflicts of interest leading to an increase in spinal fusions and bias in the doctors’ decision making process.

Allegations of financial conflicts of interest and the fact that there isn’t sufficient scientific evidence to support the use of spinal fusion as a modality of treatment for chronic low back pain, should make us reconsider our indications for spinal fusion. The decision to do spinal fusion in the absence of clear indications may have future medico-legal implications and the conflicts of interest involved may run afoul of existing legislations in some countries.
